# Thymol Reduces *agr*-Mediated Virulence Factor Phenol-Soluble Modulin Production in *Staphylococcus aureus*

**DOI:** 10.1155/2022/8221622

**Published:** 2022-05-09

**Authors:** Harshad Lade, Sung Hee Chung, Yeonhee Lee, Bajarang Vasant Kumbhar, Hwang-Soo Joo, Yun-Gon Kim, Yung-Hun Yang, Jae-Seok Kim

**Affiliations:** ^1^Department of Laboratory Medicine, Kangdong Sacred Heart Hospital, Hallym University College of Medicine, Seoul 05355, Republic of Korea; ^2^Department of Biotechnology, College of Engineering, Duksung Women's University, Seoul 01369, Republic of Korea; ^3^Department of Biological Sciences, Sunandan Divatia School of Science, NMIMS University, Mumbai 400056, India; ^4^Department of Chemical Engineering, Soongsil University, Seoul 06978, Republic of Korea; ^5^Department of Biological Engineering, College of Engineering, Konkuk University, Seoul 05029, Republic of Korea

## Abstract

*Staphylococcus aureus* is a major human bacterial pathogen that carries a large number of virulence factors. Many virulence factors of *S. aureus* are regulated by the accessory gene regulator (*agr*) quorum-sensing system. Phenol-soluble modulins (PSMs) are one of the *agr*-mediated virulence determinants known to play a significant role in *S. aureus* pathogenesis. In the present study, the efficacy of thymol to inhibit PSM production including *δ*-toxin in *S. aureus* was explored. We employed liquid chromatography–mass spectrometry (LC–MS) to quantify the PSMs*α*1–PSM*α*4, PSM*β*1 and PSM*β*2, and *δ*-toxin production from culture supernatants. We found that thymol at 0.5 MIC (128 *μ*g/mL) significantly reduced the PSM*α* and *δ*-toxin production in *S. aureus* WKZ-1, WKZ-2, LAC USA300, and ATCC29213. Downregulation in transcription by quantitative real-time (qRT) PCR analysis of response regulator *agrA* and receptor histidine kinase *agrC* upon 0.5 MIC thymol treatment affirmed the results of LC–MS quantification of PSMs. *In silico* molecular docking analysis demonstrated the binding affinity of thymol with receptors AgrA and AgrC. Transmission electron microscopy images revealed no ultrastructural alterations (cell wall and membrane) in thymol-treated WKZ-1 and WKZ-2 S*. aureus* strains. Here, we demonstrated that thymol reduces various PSM production in *S. aureus* clinical isolates and reference strains with mass spectrometry.

## 1. Introduction

*Staphylococcus aureus* is a major human bacterial pathogen associated with hospital-acquired infections and the leading cause of community-associated infections [1]. *S*. *aureus* causes many infections, including skin and soft tissue infections, osteomyelitis, bacteremia, abscesses, endocarditis, and septicemia [2]. To invade and survive in the host, *S. aureus* produces a large arsenal of virulence factors such as gene products involved in adhesion, toxins secretion, and host defense evasion [3]. These virulence factors help *S. aureus* to survive and persist in stressful *in vivo* conditions, although these are not essential for cell growth. This has led to the search for agents to inhibit virulence factors without imposing selective pressure for the development of resistance.

The expression of several *S. aureus* virulence factors including phenol-soluble modulins (PSMs) is mainly regulated by the accessory gene regulator (*agr*) quorum-sensing system in a response to cell density [4–6]. The *agr*-system is two-component signaling (TCS) transduction system comprising membrane-bound receptor histidine kinase AgrC and cytoplasmic response regulator AgrA [7]. To begin with the transcription and translation of the *agr* operon, AgrB modifies and secretes AgrD to produce autoinducing peptides (AIP). When the extracellular AIP concentration reaches a critical threshold value, the signal is sensed by AgrC, resulting in autophosphorylation of the cytoplasmic domain of AgrC followed by transfer of phosphate to AgrA. Upon phosphorylation, AgrA binds to the *P2* and *P3* promoters of *agr* operon, driving expression of the RNAII and RNAIII transcripts, respectively. Furthermore, AgrA directly binds to the promoters for transcription of the PSMs in an RNAIII-independent fashion [8, 9]. The *P2* promoter drives a positive feedback loop resulting in the upregulation of *agr* operon, whereas the P3 promoter drives the transcription of RNAIII, the effector molecule of *agr* operon [9–11]. The RNAIII is responsible for the upregulation of extracellular proteins such as *α*-hemolysin, enterotoxins, leukocidins, lipases, and proteases along with the downregulation of cell-surface proteins such as Protein A and fibronectin-binding proteins [9]. Furthermore, the *hld* gene is located on the RNAIII portion of the *agr* operon, which encodes for *δ*-toxin [9, 11]. As the *agr-*system is central to the expression of several virulence factors including PSM production, it has often been proposed as a potential target to attenuate *S. aureus* pathogenicity.

PSMs are a group of small amphipathic peptides, including PSM*α*1 to PSM*α*4 (~20–25 amino acids), PSM*β*1 to PSM*β*2 (~45 amino acids), and *δ*-toxin (~26 amino acid) [8, 12–15]. The *α*PSMs possess the most strong cytolytic activity among PSMs [8]. *δ*-toxin is amphipathic and alpha-helical in structure and is generally the most strongly expressed peptide than other PSMs. It possesses moderate cytolytic capacities and the capacity to stimulate formyl peptide receptor 2 (FPR2) [14, 15]. PSM peptides are involved in a series of biological functions critical for staphylococci pathogenesis [8, 16] and may cause lysis of human erythrocytes and leukocytes and inflammatory response stimulation [17]. PSMs can aggregate and form bacterial functional amyloids [18], which are speculated to contribute to biofilm structuring and detachment [16]. Biofilm-associated *S. aureus* infections resist antimicrobial treatment and innate host immune response [19]. This requires aggressive antimicrobial therapy and the removal of infected tissues [20].

An alternative strategy that is currently being widely investigated to tackle antimicrobial-resistant staphylococcal infections includes antivirulence therapy [21]. Numerous natural compounds inhibiting virulence factor production of *S. aureus*, either alone or in combination with traditional antibiotics, have been reported [21]. For example, thymol (2-isopropyl-5-methylphenol), a constituent of thyme herb (*Thymus vulgaris* L.), possesses a wide spectrum of antimicrobial activity [22–29] and reduces the biofilm formation of *S. aureus* strains [30–33]. Furthermore, it is known to inhibit staphyloxanthin production in MRSA [34]. It decreases the production of *α*-hemolysin and enterotoxins (i.e., *sea* and *seb*) in both methicillin-sensitive *S. aureus* (MSSA) and MRSA strains in a dose-dependent manner [26]. However, no report is available for the PSM inhibitory activity of thymol. Hence, the present study is aimed to explore the inhibitory potential of thymol on the PSMs and *δ*-toxin in different *S. aureus* strains and understand the mechanisms underlying its action.

## 2. Materials and methods

### 2.1. Bacterial Strains and Growth Conditions

The *S. aureus* strains used in this study are described in Table 1. The clinical isolates of *S. aureus* WKZ-1 and *S. aureus* WKZ-2 are isogenic strains except for the presence of methicillin resistance Staphylococcal cassettes chromosome *mec* (SCC*mec*) in WKZ-2 [35–37]. *S. aureus* Los Angeles County (LAC) of pulsed-field type USA300 [38] and its isogenic *Δagr* (*agr* system entirely deleted except for a 3′ part of RNAIII) and *Δ3KO* (*αpsm*, *βpsm*, and *hld* knockout) were also evaluated [39, 40]. The reference strains of *S. aureus* ATCC29213 and *S. aureus* RN4220 were obtained from the American Type Culture Collection (ATCC) and BEI Resources, respectively. For the PSM production assay, the *S. aureus* strains were grown in tryptic soy broth (TSB) (BD, Sparks, MD) at 37°C with shaking (200 rpm). The bacterial stock cultures were stored in skimmed milk at -70°C.

### 2.2. Minimum Inhibitory Concentration (MIC) Determination

The MIC of thymol (CAS No. 89-83-8; Sigma-Aldrich, St. Louis, MO) against *S. aureus* strains was determined by broth microdilution assay following the Clinical and Laboratory Standards Institute (CLSI) guidelines [41]. Cation-Adjusted *Mueller Hinton* II *Broth* (CA-MHB) (BD, Sparks, MD) was used for the estimation of MIC, as recommended by the CLSI [42]. A stock solution of thymol (51.2 mg/mL) was prepared in dimethyl sulfoxide (DMSO) (Sigma-Aldrich) and working solutions (2–1024 *μ*g/mL) were prepared by serial twofold dilutions in CA-MHB. The working solution was then added in polystyrene 96-well microtiter plate-U bottoms (FALCON, Corning, NY) with a final assay volume of 100 *μ*L per well. A suspension of *S. aureus* strains was prepared in CA-MHB and inoculated into each well of the microtiter plate to give a final cell density of 5 × 10^5^ colony-forming units (CFU)/mL. The plates were incubated at 37°C for 24 h and the MIC values were recorded as the lowest concentration of thymol with no visible growth. *S. aureus* ATCC29213 was used as a quality control strain for MIC testing.

### 2.3. PSM Quantification by Liquid Chromatography–Mass Spectrometry (LC–MS)

The PSM production by *S. aureus* strains was quantified by LC–MS as described previously with some modifications [43, 44]. Briefly, overnight grown *S. aureus* strains (30 *μ*L) were inoculated in 3 mL of TSB (with and without 0.5 MIC thymol) and incubated at 37°C under shaking conditions (200 rpm) for 20 h [45]. The cultures were centrifuged at 4,000 rpm for 20 min at 4°C to pellet the cells and supernatant was used for PSM quantification. *S. aureus* LAC USA300 strain was employed as a positive control for PSMs quantification, while its isogenic mutant *Δ3KO* was used as negative controls.

For LC–MS analysis, 5 *μ*L of supernatant was injected into the C8 (ZORBAX SB-C8, 2.1 × 5 mm, 1.8 *μ*m) (Agilent, Santa Clara, CA) column connected to a Waters ZQ 2000 LC–MS system (Waters, Milford, MA) and eluted by a gradient program with trifluoroacetic acid (TFA; 0.05%) in water and 0.05% TFA in acetonitrile at a flow rate of 0.3 mL/min. Electrospray ionization of samples was performed at 3.5 kV, and ions were infused into the ion entrance of a mass spectrometer. The *m*/*z* values of the analytes were scanned continuously, and mass spectra were recorded. The *m*/*z* values of 2+ and 3+ charged ions of *α*-type PSMs and 3+ and 4+ charged ions of *β*-type PSMs were used to extract chromatograms for quantification of each PSM [43]. PSMs were quantified by integration of the extracted ion chromatogram of **formyl- and deformylated-PSMs**. The concentration of PSMs was determined by calibration with three different concentrations of each synthetic formyl PSM. Formyl PSMs were synthesized by Peptron (Daejeon, Korea) and Cosmogenetech (Daejeon, Korea).

### 2.4. Quantitative Real-Time (qRT) PCR Analysis

To assess the effect of thymol on the expression of genes associated with PSM production, qRT-PCR was performed. The *S. aureus* strains were cultivated in TSB (with and without 0.5 MIC thymol) under similar conditions as the PSMs quantification assay. After 6 h of growth, the bacterial cells were harvested by centrifugation at 5,000x*g* for 10 min, and pellets were resuspended in RNAprotect Bacteria Reagent (Qiagen, Düsseldorf, Germany) and incubated for 5 min at room temperature. Cells were pelleted by centrifugation at 5,000x*g* for 10 min, RNAprotect Bacteria Reagent was discarded, and the samples were stored at −80°C.

RNA extraction was carried out using the RNeasy Plus Mini Kit (Qiagen, Düsseldorf, Germany) with initial lysis in 1 mg/mL lysostaphin solution (Sigma-Aldrich, St. Louis, MO) at 37°C for 30 min. RNA concentration was analyzed using a NanoDrop 1000™ spectrophotometer (Thermo Fisher Scientific, Wilmington, DE). The PrimeScript™ RT Master Mix and TB Green™ Fast qPCR Mix kits (Takara, Tokyo, Japan) were used for RNA reverse transcription and qPCR system preparation separately. Real-time PCR was performed on a LightCycler® 480 RT-PCR system (Roche, Mannheim, Germany) with specific primers (Table 2). RT-PCR conditions were initial denaturation (95°C for 5 sec), followed by denaturation (95°C for 10 sec), annealing (58°C for 10 sec), and extension (72°C for 10 sec) for 45 cycles. Relative gene expression was calculated by the 2^−*ΔΔ*CT^ method with housekeeping gene *gyrB* as an internal control [46].

### 2.5. Molecular Docking Analysis

To explore the binding mode and interaction of thymol with AgrA and AgrC of *S. aureus*, molecular docking was performed using AutoDock4.2 software [49]. The crystal structures of AgrA (PDB ID: 3BS1) and AgrC (PDB ID: 4BXI) were retrieved from the Protein Data Bank (http://www.rcsb.org). The missing residues of AgrC were modelled using the SWISS-MODEL server (https://swissmodel.expasy.org/) [50] and further energy minimization was performed using the GROMACS 2021.1 (https://www.gromacs.org) to obtain the least energy conformation of AgrC. The AgrC contains ATP binding domain [51]; hence, ATP was docked using AutoDock4.2 [49]. The purpose of using AgrC-ATP complex for docking study was to understand the binding mode of thymol. These AgrA and AgrC were further used for molecular docking study of thymol (PubChem ID: 6989) as well as previously reported antivirulence compounds. The savirin (PubChem ID: 3243271), staquorsin, and bumetanide (PubChem ID: 2471) were used as a positive control for AgrA [52–54]. The atomic coordinates of staquorsin were built using Discovery Studio Visualizer 2016 (BIOVIA, Dassault Systèmes, San Diego). The binding mode of thymol AgrC was determined through a blind docking approach followed by a local docking protocol (http://autodock.scripps.edu) using the Autodock4.2 software. However, the binding mode of thymol as well as savirin, staquorsin, and bumetanide with AgrA was investigated using a site-specific local docking approach considering the kinase domain, similar to earlier studies [52, 53]. The least binding energy docked conformation of the above-mentioned compounds with AgrA and AgrC was further analyzed and visualized through the PyMol (The PyMOL Molecular Graphics System, Version 2.0 Schrödinger, LLC) and Discovery Studio Visualizer 2016.

### 2.6. Minimal Biofilm Inhibitory Concentration (MBIC) Assay

The antibiofilm activity of thymol against *S. aureus* strains was evaluated by MBIC assay. MBIC assay was performed in TSB supplemented with 1.0% D-(+)-glucose (TSBg) to support biofilm formation and reproducible quantification [55]. Briefly, *S. aureus* was diluted in TSBg to make the inoculum. Thymol was dissolved in DMSO and then serially diluted in TSBg twofold across the wells of 96-well polystyrene plate with flat bottoms (FALCON, Corning, NY). The microtiter plate wells contained a total volume of 200 *μ*L TSBg containing the bacterial inoculum (1 × 10^6^ CFU/mL) and thymol (32–256 *μ*g/mL). After incubation at 37°C for 24 h in stationary conditions, the bacterial culture from the microtiter plate well was gently aspirated and washed twice with 200 *μ*L of phosphate-buffered saline (PBS, pH 7.4) to remove nonadherent bacteria. The adherent bacteria were fixed by heating at 65°C for 1 h and were stained with 150 *μ*L of 0.1% (*w/v*) crystal violet (Sigma-Aldrich, St. Louis, MO) for 5 min [56]. The excess crystal violet stain was then discarded, and the plates were washed twice with 200 *μ*L per well of PBS to remove the nonadherent dye and allowed to dry for 30 min at room temperature. The stained adherent biofilm was dissolved in 150 *μ*L per well of 33.0% glacial acetic acid (v/v) for 30 min, and MBIC was determined by measuring the OD_595_ on MULTISKAN FC reader (Thermo Fisher Scientific). The percentage biofilm inhibition was calculated using the formula [32, 48]:

(1) $$\begin{array}{c} \text{Biofilm inhibition }\left(\%\right)=\left[\frac{\text{Control OD}-\text{Treated OD}}{\text{Control OD}}\right]\text{X }100. \end{array}$$

A well-characterized biofilm-producing strain *S. aureus* RN4220 was employed as a positive control [55, 57], while uninoculated culture media served as a negative control.

### 2.7. Transmission Electron Microscopy (TEM) Analysis

TEM was carried out to investigate the effects of 0.5 MIC thymol on the *S. aureus* ultrastructure as described previously [58, 59]. Briefly, 3 mL of TSBg (with and without 0.5 MIC thymol) in a 6-well plate (SPL Life Sciences, Pocheon, Korea) inoculated with *S. aureus* WKZ-1 and *S. aureus* WKZ-2 cultures (1 × 10^6^ CFU/mL) was incubated for 24h at 37°C. The culture broth was gently aspirated, and cells were washed with PBS (pH 7.4), fixed with 2.5% (v/v) glutaraldehyde, and postfixed with 1.0% osmium tetroxide (OsO_4_) in sodium cacodylate buffer (pH 6.5; 50 mM). Samples were then progressively dehydrated with 15 min treatments of increasingly concentrated ethanol (50%, 70%, 90%, 95%, and 100%). After dehydration, the bacterial samples were dried with hexamethyldisilazane (HMDS), embedded in Epon 82 (*Ted Pella*, Redding, CA), and sectioned into 70 nm slices using a Leica Ultracut UCT ultramicrotome. The sections were then stained with uranyl acetate and lead citrate. Morphological and ultrastructural alterations of cells were observed and photographed using a Cryo TEM with a field-emission gun at 200 kV of FEI Tecnai F20G2 (Thermo Fisher Scientific). The TEM analysis was performed at the Advanced Analysis Center, Korea Institute of Science and Technology (KIST), Seoul, Korea.

### 2.8. Statistical Analysis

Statistics were determined using GraphPad Prism (version 9.2.0) and Microsoft Excel. All the assays were performed in replicate and the results were presented as the mean ± standard deviation (SD). The statistical significance was determined by an unpaired Student's *t*-test and one-way analysis of variance (ANOVA) followed by Dunnett's multiple comparisons test. *P* values <0.05 were considered significant.

## 3. Results

### 3.1. MIC of Thymol against *S. aureus* Strains

The MIC values of thymol as determined by CLSI guidelines against *S. aureus* WKZ-1 and WKZ-2 clinical isolates, as well as reference strain LAC USA300 and its isogenic mutants (*Δagr* and *Δ3KO*), ATCC29213, and RN4220, were 256 *μ*g/mL. Notably, the MIC did not change against MRSA strains such as WKZ-2 and LAC USA300.

### 3.2. Thymol Reduces PSM Production by *S. aureus* Clinical Isolates and Reference Strains

The bioactivity of thymol was tested at 0.5 MIC via an *in vitro* assay that evaluated its ability to inhibit PSM production. To ensure that 0.5 MIC thymol reduces PSM production in *S. aureus* strains without growth attenuation, the growth was measured as OD_595_ after 20 h incubation at 37°C (Figure S1). The results suggest that 0.5 MIC thymol did not inhibit the growth of WKZ-1 and WKZ-2 as well as all the reference strains and mutants.

**Figure 1:**
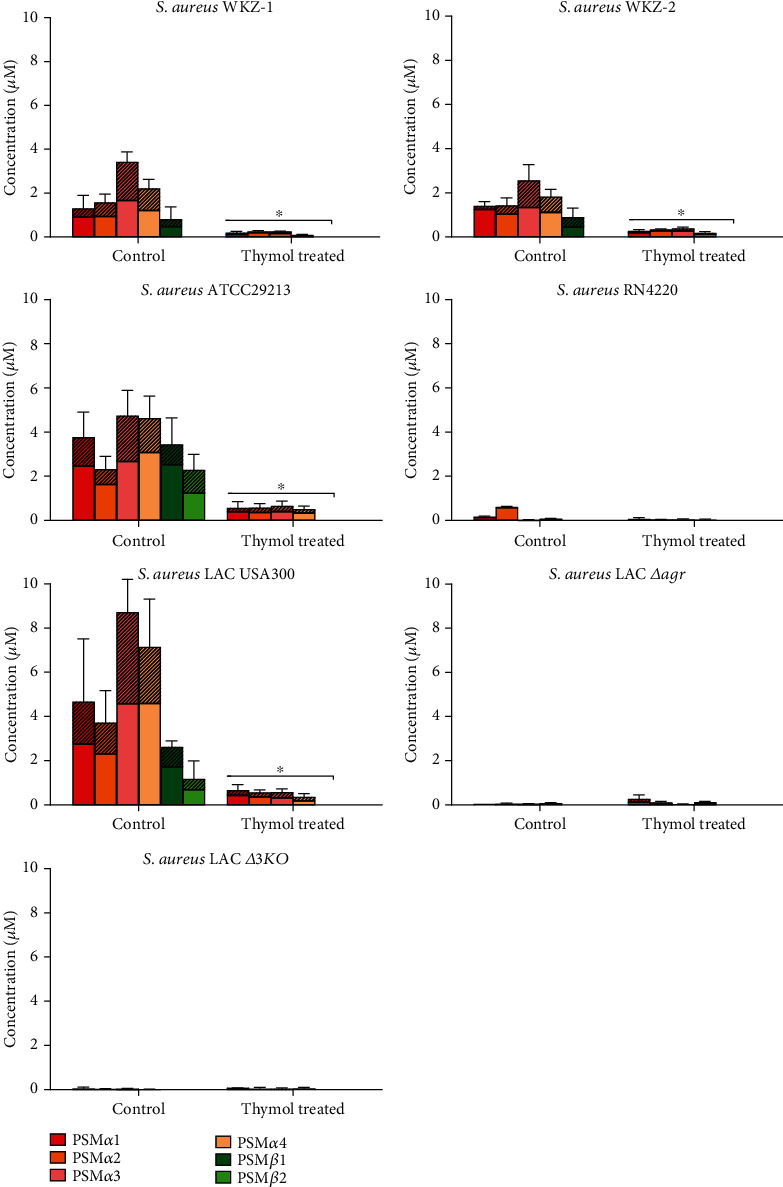
Production of *α*PSMs and *β*PSMs by *S. aureus* strains cultured in TSB (with and without 0.5 MIC thymol) for 20 h. PSMs concentrations in the culture supernatant were measured by LC–MS. Values represent means ± SD of three independent experiments. Striped portions of bars represent deformylated form of PSMs.

Mass spectrometric analysis revealed the significantly reduced production of PSM*α*1, PSM*α*2, PSM*α*3, and PSM*α*4 in WKZ-1 and WKZ-2 isolates after 0.5 MIC thymol treatment (*P* < 0.05) (Figure 1). Furthermore, a significant reduction in the productions of PSM*α*1-*α*4 was also observed in ATCC29213 and LAC USA300 culture supernatants (*P* < 0.05). LAC *Δagr* and *Δ3KO* did not produce *α*PSMs.

In this study, WKZ-1 and WKZ-2 isolates produced a considerable amount of PSM*β*1 and subsequent 0.5 MIC thymol treatment significantly reduced its levels (*P* < 0.05) (Figure 1). Furthermore, thymol reduced the production of PSM*β*1 and PSM*β*2 in reference strains of ATCC29213 and LAC USA300 (*P* < 0.05). No production of *β*PSMs was observed in the RN4220 strain.

**Figure 2:**
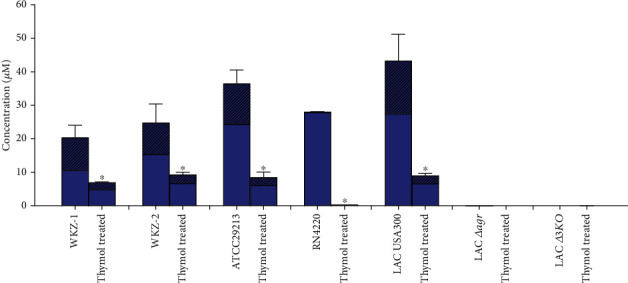
Production of *δ*-toxin by *S. aureus* strains cultured in TSB (with and without 0.5 MIC thymol) for 20 h. *δ*-toxins concentration in the culture supernatant was measured by LC–MS. Values represent means ± SD of three independent experiments. Striped portions of bars represent deformylated form of *δ*-toxins.

We observed that 0.5 MIC thymol significantly reduced the *δ*-toxin production in WKZ-1 and WKZ-2 isolates as well as ATCC29213, RN4220, and LAC USA300 (*P* < 0.05) (Figure 2). As shown in Figure 2, LAC *Δagr* and *Δ3KO* did not produce *δ*-toxin. Together, these results demonstrate that thymol is effective in reducing the PSMs and *δ*-toxin production of *S. aureus*.

### 3.3. Thymol Target *agrA* and *agrC* of *S. aureus*

**Figure 3:**
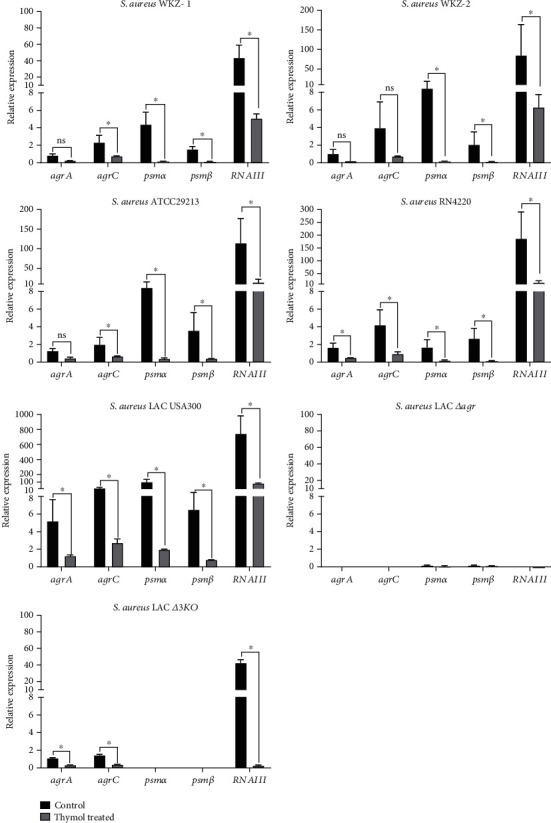
Relative change in expression of genes associated with PSM production in *S. aureus* strains cultured in TSB (with and without 0.5 MIC thymol). The *gyrB* was used as a housekeeping gene. Error bars indicate SD. The asterisks represent statistical significance (*P* ≤ 0.05), compared with the same genes in the control.

With the finding that thymol reduces PSM production, we focused on important *agr*-system genes that are known to regulate PSM production in *S. aureus*. The expression of all candidate genes was analyzed from the PSM production assay after 6 h. As shown in Figure 3, 0.5 MIC thymol treatment reduced the expression of the regulator genes of *agrA* (response regulator) and *agrC* (receptor histidine kinase) in WKZ-1 and WKZ-2. Furthermore, ATCC29213, RN4220, LAC USA300, and LAC *Δ3KO* also showed the downregulation of *agrA* and *agrC* after thymol treatment. No expression of *agrC* and *agrA* genes was observed in the LAC *Δagr* mutant.

The expression levels of *psmα*, *psmβ*, and *RNAIII* (effector molecule of *agr*-system) were significantly downregulated in WKZ-1 and WKZ-2 as well as ATCC29213, RN4220, and LAC USA300 after 0.5 MIC thymol treatment (*P* < 0.05) (Figure 3). No expression of *psmα*, *psmβ*, and *RNAIII* genes was observed in LAC *Δagr* as expected. Because LAC *Δ3KO* mutant only has a start codon change from ATG to ATT, the *hld* gene was still detected but not functional.

### 3.4. Binding Mode of Thymol with AgrA and AgrC Regulator

**Figure 4:**
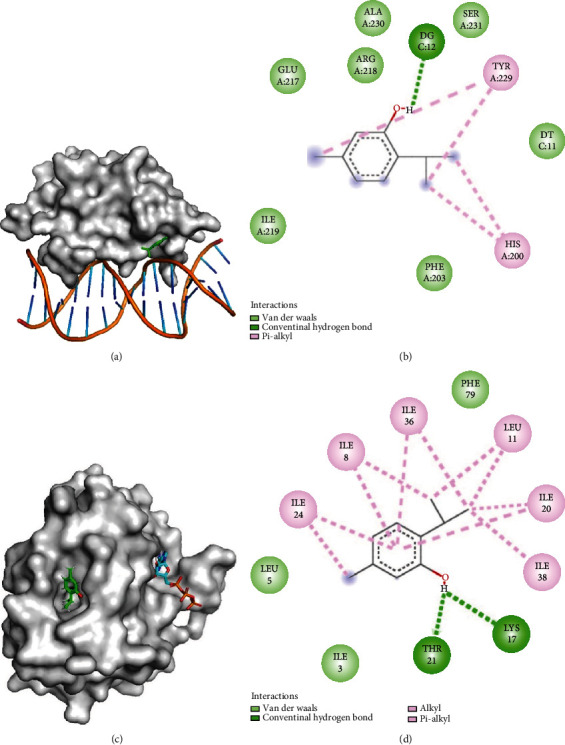
Binding mode of AgrA and AgrC with thymol using molecular docking. Here, AgrA and AgrC are shown in the space fill model with gray color, while thymol is shown in the stick model with carbon in green and oxygen in red color. The ATP in AgrC is shown in the stick model and carbon in cyan, oxygen in red, and phosphorus in golden color. (a) Binding mode of thymol with AgrA at kinase domain. (b) 2D interactions of thymol with AgrA atoms. (c) The interactions of thymol with AgrC. (d) Interaction network of thymol with AgrC residues. Panel (b) and (d) show the residues with dark green color form conventional hydrogen bonding, light green form van der Waals forces, and pink form alkyl type of interactions with thymol.

Results of the molecular docking study showed that thymol interacts with AgrA and AgrC of *S. aureus*. The least energy docked conformation of thymol was found to be -4.31 and -5.13 kcal/mol with AgrA and AgrC, respectively (Table 3). The AgrA-thymol complex (Figure 4(a)) was stabilized by the hydrogen bonding interactions with Glu217 (2.1 Å), His200 (2.5 Å), and nucleotide G12 (1.8 Å) (Figure 4(b) and Table 3). Here, thymol forms van der Waals interaction with Glu217, Arg218, Ala230, Ser231, Phe203, and *π*-alkyl type of interactions with Tyr229 and His200. Furthermore, the control docking studies with savirin, staquorsin, and bumetanide reveal the considerable binding affinity with AgrA (Table 3). The least binding energy conformation of savirin, staquorsin, and bumetanide was found to be -6.40, -6.83, and -4.06 kcal/mol, respectively. We found that AgrA-savirin complex (Figure S2a) was stabilized by bonding interactions with the Glu217 (1.7 Å), His200 (1.8 Å), and *π*-*π* type of interaction with Tyr229 (Figure S2b and Table 3), similar to an earlier study [52]. Furthermore, AgrA-staquorsin complex (Figure S2c) was stabilized by bonding interactions with Ser202 (1.9 Å), His200 (2.1 Å), nucleotide Adenosine (1.6 Å), and nucleotide Thymin (1.6 Å), and carbon-hydrogen interaction with the Glu217 (1.9 Å) (Figure S2d and Table 3). Staquorsin also forms van der Waals, *π*-carbon, *π*-anion, *π*-sulfur, *π*-alkyl, and *π*- *π* type of interactions with AgrA. The AgrA-bumetanide complex (Figure S2e) shows the hydrogen interaction with Glu217 (2.0 Å), Ala230 (2.7 Å), and DC (1.8 Å) (Figure S2f and Table 3).

Analysis of the AgrC-thymol complex (Figure 4(c)) showed that it was stabilized by the hydrogen bonding interaction with Lys17 (2.72 Å) (Figure 4(d) and Table 3). Additionally, Ile24, Ile8, Leu11, Ile20, and Ile36 make alkyl types of interactions, while Ile8, Ile20, Ile24, and Ile36 make an *π*-type of interactions with thymol. Here, thymol shows a significant binding affinity with the AgrC-ATP complex and may inhibit the dephosphorylation ATP to ADP and Pi. This may lead to the unavailability of Pi for activation of AgrA.

### 3.5. Antibiofilm Potential of Thymol against *S. aureus* Strains

**Figure 5:**
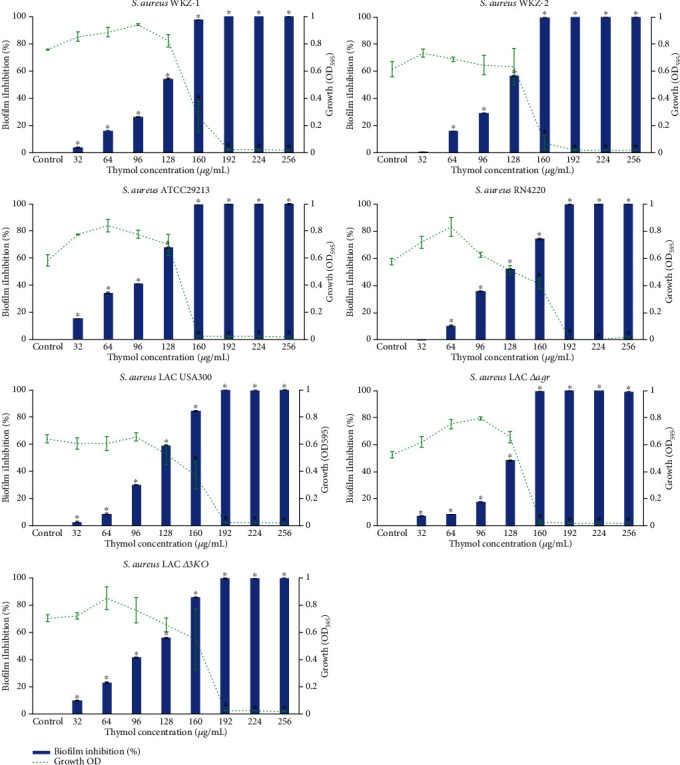
Effect of thymol at various concentrations (32-256 *μ*g/mL) on growth and biofilm formation of *S. aureus* strains. The line graph represents the growth while the bar graph represents the percentage of biofilm inhibition. Error bars represent SD and asterisk indicates statistical significance (*P* ≤ 0.05).

The effect of thymol at increasing concentrations (32 to 256 *μ*g/mL) on biofilm formation by *S. aureus* strains was assessed on polystyrene surface. The growth OD of control and thymol-treated *S. aureus* strains did not show any significant difference up to 128 *μ*g/mL of thymol concentrations (*P* < 0.05) (Figure 5). At 128 *μ*g/mL concentration, thymol showed maximum of biofilm inhibition in all strains including *S. aureus* WKZ-1 (54.3%), *S. aureus* WKZ-2 (56.7%), *S. aureus* ATCC29213 (67.8%), RN4220 (74.4%), and LAC USA300 (58.9%) and its isogenic *Δagr* (48.4%) and *Δ3KO* (55.8%) (*P* < 0.05). Biofilm inhibition beyond 128 *μ*g/mL may appear due to growth inhibitory effects of thymol.

### 3.6. Morphological Changes by TEM

**Figure 6:**
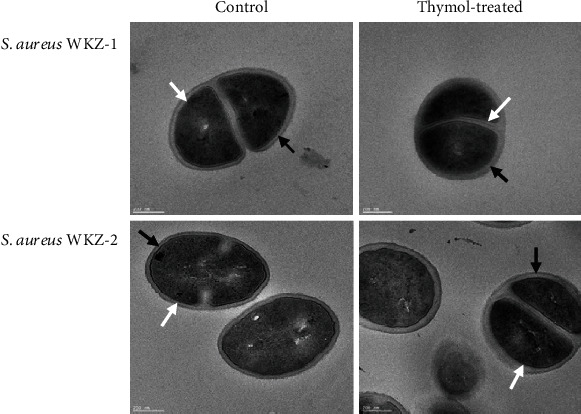
TEM images of the ultrastructure of *S. aureus* WKZ-1 and *S. aureus* WKZ-2 control and 0.5 MIC thymol-treated cells. The cell wall (black arrow), cell membrane (white arrow), and septa were visible with thymol-treated bacterial cells, similar to the control. No disruption of the cell wall or cell membrane was observed following thymol treatment. Scale bar represents 200 nm.

TEM was used to observe changes to the cell structure after 0.5 MIC thymol treatment. The TEM images confirmed that WKZ-1 and WKZ-2 cells were intact after treatment with a subinhibitory concentration of thymol (Figure 6). Moreover, TEM images of the treated *S. aureus* strains confirmed intact septa. These findings suggest that the integrity of *S. aureus* cells was maintained with 0.5 MIC thymol treatment with no destruction of the cell wall and cell membrane morphologically.

## 4. Discussion

The *agr*-system plays an important role in the regulation of several virulence factors in *S. aureus*, such as upregulation of PSMs, *δ*-toxin, nucleases, lipase, and other staphylococcal toxins [9, 39]. Thus, inhibition of the *agr*-system has been suggested as a target for controlling *S. aureus* virulence [60, 61]. Thymol, a herb-derived essential oil, has been reported to inhibit the *agr*-mediated virulence factor of *α*-hemolysin in the MRSA strain 2985 [26]. However, the previous reports were performed for a quite limited number of *S. aureus* strains and without direct measurement of PSM*α*1-4, PSM*β*1-2, and *δ*-toxin production by mass spectrometry. In addition, a previous study showed the inhibitory effect of thymol on master regulator *agrA* expression [26], while another study showed an unaltered expression of *agrA* [32].

In the present study, we found a significant reduction in the production of PSM*α*1-*α*4 in both MSSA (WKZ-1) and MRSA (WKZ-2) clinical isolates by 0.5 MIC thymol treatment (Figure 1). PSM peptides are produced as functional amyloids that play distinct roles in *S. aureus* pathogenicity [62], and its inhibition in both MSSA and MRSA strains indicates the antivirulence potential of thymol. Consistent with previous studies, we found *δ*-toxin was the most strongly produced peptide in WKZ-1 and WKZ-2 as well as other *S. aureus* strains (Figure 2) [14, 15]. *δ*-toxin possesses a moderate capacity to lyse human neutrophils and PSM-mediated phenotypes like bacteremia [8, 13].

To understand the mechanism of PSMs reduction by thymol, the gene expression analysis by qRT-PCR and in silico molecular docking studies of major PSMs regulators (AgrA and AgrC) were performed. qRT-PCR analysis showed downregulation of *agrA* and *agrC* upon thymol treatment (Figure 3), which could reduce PSM production. We found a decrease in the *agrC* expression in 0.5 MIC thymol-treated *S. aureus* cultures (Figure 3) in contrast to the unaltered expression of *agrC* previously observed in *S. aureus* Newman strain [32]. We observed that *S. aureus* LAC *Δagr* mutant did not produce *α*PSMs, *β*PSMs, and *δ*-toxin as expected. Notably, previous studies reported the *agr*-system as the therapeutic target to attenuate *S. aureus* virulence [52, 63]. A functional *agr*-system is essential for *S. aureus* virulence as shown by the reduction of pathogenicity in isogenic *agr* mutants [39, 64, 65].

We found the transcript levels of *RNAIII* encoding *δ*-toxin were significantly reduced in all *S. aureus* strains upon thymol treatment (Figure 3). *δ*-toxin is a member of the PSMs family encoded by the *hld* gene, which is located on the RNAIII portion of the *agr* operon [8, 13]. As a key effector molecule of the *agr*-system, *RNAIII* is associated with the expression of several virulence genes in *S. aureus* [66]. The RNAIII inhibiting peptide (YSPWTNF-NH2) and its synthetic analogs were reported to inhibit *RNAII* and *RNAIII* transcription as well as effectively suppress diseases caused by *S. aureus* [67]. Thus, inhibition of *RNAIII* gene expression by thymol might be an effective strategy for reducing the production of *δ*-toxin as well as other virulence factors.

**Figure 7:**
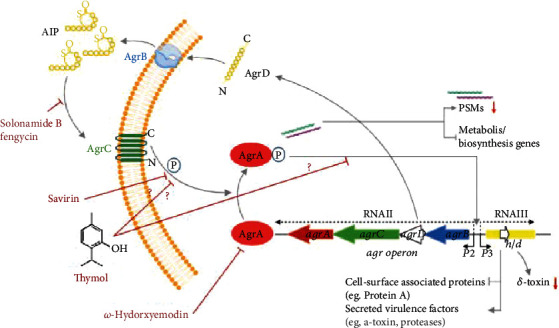
Schematic of the *S. aureus agr* quorum-sensing system. The binding affinity of thymol with AgrC could affect the conformational properties of AgrC essential for dephosphorylation of ATP to ADP + Pi, leading to interference with AgrA activation. Thymol may also block the transcription function of AgrA, leading to inhibition of PSM production. Red arrows show inhibition of PSMs including *δ*-toxin production in *S. aureus*. The agents targeting *agr*-mediated virulence of *S. aureus* are shown: solonamide B, and fengycin (competitively interferes with AIP binding to AgrC) [44, 68, 69], savirin (inhibit AgrC and AgrA downstream of AIP sensing) [52], *ω*-hydroxyemodin (directly binds to AgrA and prevents the interaction of AgrA with *P2* promoter) [70].

The molecular docking study showed the significant binding efficacy of thymol with AgrA and AgrC regulators (Table 3). Thymol formed conventional hydrogen bonding, alkyl, and *π*-type of interactions with AgrA and AgrC. Interestingly, thymol prefers a similar binding mode that interferes with AgrA-DNA binding as reported previously for savirin [52]. The binding of thymol to the AgrA may cause the inhibition of AgrA−*P2/P3* interactions, leading to the inhibition of *agr*-mediated virulence factor PSM production. It is reported that savirin disrupts *S. aureus agr*-system by inhibiting the activation of AgrA, thus preventing the upregulation of virulence genes [52]. Our model analysis showed consistent results with the previous reports on savirin, staquorsin, and bumetanide [52–54], and thymol also exhibited significant binding efficiency to AgrA of *S. aureus*. We speculate that the binding affinity of thymol with AgrC may also affect the conformational properties of AgrC essential for dephosphorylation of ATP to ADP + Pi, leading to interference with AgrA activation due to the unavailability of Pi group (Figure 7). Thymol could also interfere with the *agr*-system by blocking the transcription function of AgrA.

In this study, the inhibition of *S. aureus* biofilms by thymol was found to be concentration-dependent, which is consistent with previous studies [30–32]. Biofilm formation in *S. aureus* is associated with antimicrobial resistance [19, 71], and inhibition of biofilm formation could be a promising strategy against *S. aureus* infections. This study showed the inhibition of PSMs and *δ*-toxin with the hindering biofilm formation of *S. aureus* by thymol, and these results suggest potential and additive advantages of thymol against *S. aureus* infections.

## 5. Conclusion

Antimicrobial strategies targeting virulence factors have attracted great interest recently. The present study revealed the antivirulence potential of thymol, especially PSMs and *δ*-toxin of *S. aureus* by inhibiting *agr*-mediated virulence factors. Thymol, a herb-derived molecule as an antivirulence agent, could inhibit the PSM and *δ*-toxin production, suggesting the potential therapeutic agent on *S. aureus* infections.

## Figures and Tables

**Table 1:** The *S. aureus* strains used in this study.

Strain name	Details
*S. aureus* WKZ-1 (MSSA)	Clinical isolate (NR-28984)
S. aureus WKZ-2 (MRSA)	Clinical isolate (NR-28985)
*S. aureus* ATCC29213 (MSSA)	ATCC strain
*S. aureus* RN4220 (MSSA)	ATCC strain (NR-45946)
*S. aureus* LAC USA300 (MRSA)	LAC wild-type strain
*S. aureus* LAC *Δagr*	LAC *Δagr* (*agr* system entirely deleted)
*S. aureus* LAC *Δ3KO*	LAC *Δ3KO* (*psmα*, *psmβ*, and *hld* knockout)

**Table 2:** List of primers used for the qPCR analysis.

Target gene	Primer name	Sequence (5′ to 3′)	Ref.
*agr*A	*agrA*-for	ACGAGTCACAGTGAACTTAC	[47]
	*agrA*-rev	GACAACAATTGTAAGCGTGT	
*agrC*	*agrC*-for	CATTCGCGTTGCATTTATTG	[48]
	*agrC*-rev	CCTAAACCACGACCTTCACC	
*psmα*	*psmα*-for	GAAGGGGGCCATTCACAT	[47]
	*psmα*-rev	GTTGTTACCTAAAAATTTACCAAGT	
*psmβ*	*psmβ*-for	TGGAAGGTTTATTTAACGCA	[47]
	*psmβ*-rev	AAACCTACGCCATTTTCAAC	
*RNAIII*	*RNAIII*-for	TTTATCTTAATTAAGGAAGGAGTGA	[47]
	*RNAIII*-rev	TGAATTTGTTCACTGTGTCG	
*gyrB*	*gyrB*-for	ATCTGGTCGTGACTCTAGAA	[47]
	*gyrB*-rev	TGTACCAAATGCTGTGATCA	

**Table 3:** Binding energy and main interactions of thymol and positive control antivirulence compounds with AgrA and AgrC of *S. aureus*.

Protein	Ligand	Binding energy (kcal/mol)	Atoms involved in interactions	Distance (°)	Angle (°)	Fig.
AgrA	Thymol	-4.31	LIG1:H - GLY184:O HIS169:HA - LIG1:O	1.78 2.78	161.10 118.05	4b
	Savirin	-6.40	Glu217-O…NH-drug His200-N…HC-drug	1.7 1.8	130.8 158.4	S2b
	Staquorsin	-6.83	Ser202-O...HO-drug His200-N...HN-drug Glu206-O...HC-drug DT11-O….HN-drug DA-O…x..HO-drug	1.9 2.1 1.9 1.6 2.8	136.5 140.8 136.1 153.4 139.6	S2d
	Bumetanide	-4.06	Glu217O...HO-drug Ala230-NH...OP-drug DC-12-O...HO-drug	2.0 2.7 1.8	164.2 120.6 151.6	S2f
AgrC	Thymol	-5.13	Thr21-O….HO-LIG	1.9	164.8	4d

## Data Availability

The data used to support the findings of this study are shown in the manuscript.

## Supplementary Materials

Supplementary Materials The supplementary data S1 and S2 used to support the findings of this study is included within the supplementary information file.
