# Supplementary material for: Thymol Reduces agr-Mediated Virulence Factor Phenol-Soluble Modulin Production in Staphylococcus aureus
Source: Biomed Res Int. 2022 May 9;2022:8221622. doi: 10.1155/2022/8221622 (PMC9110180; doi:10.1155/2022/8221622)
Supplement: Supplementary Materials — The supplementary data S1 and S2 used to support the findings of this study is included within the supplementary information file. [file 8221622.f1.docx]

Supplementary information

Figure S1: Growth of *S. aureus* strains cultured in TSB (with and without 0.5 MIC thymol) after 20 h incubation at 37 ^o^C with shaking (200 rpm). The growth OD of control and thymol treated *S. aureus* strains did not show any significant difference.


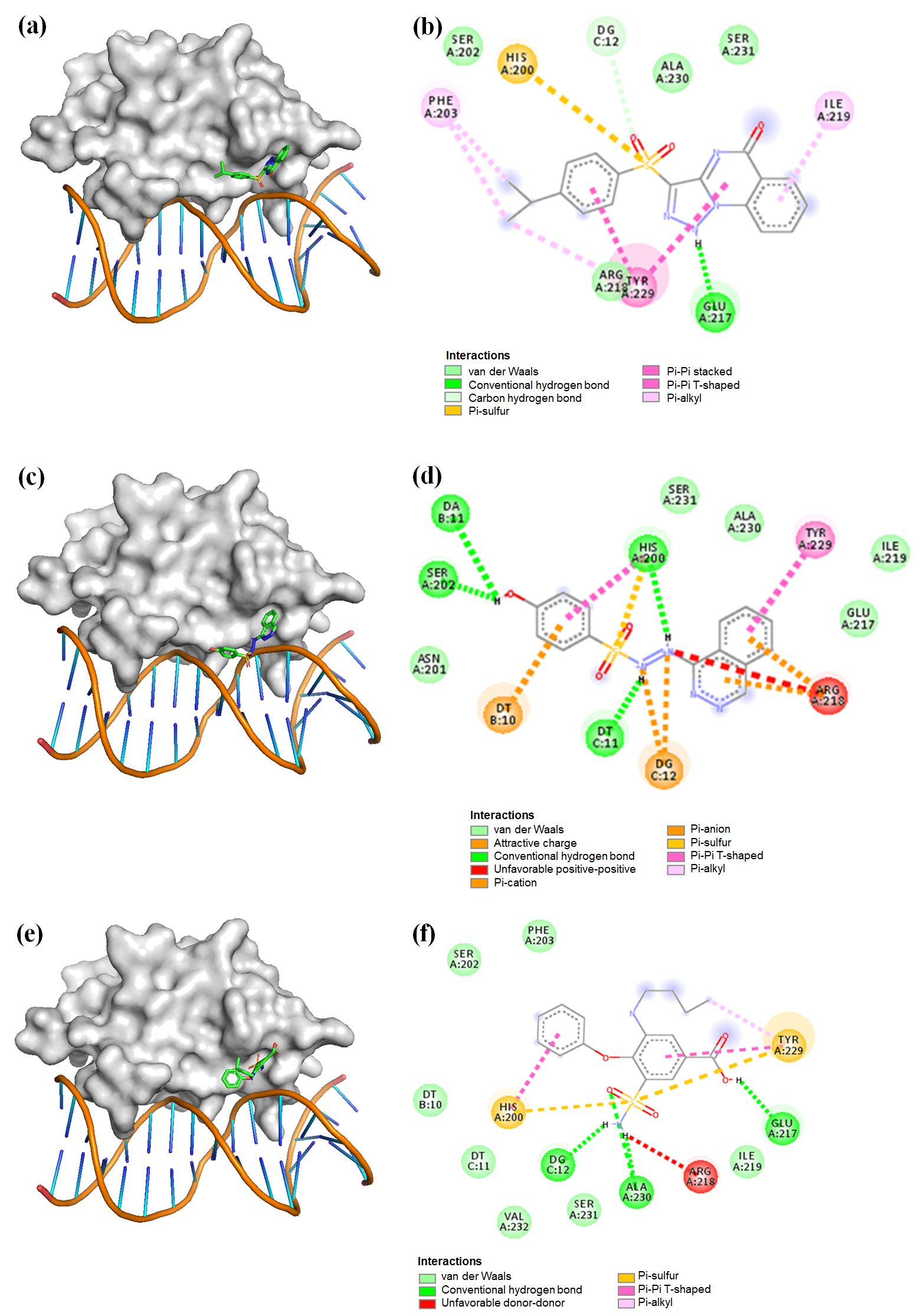


Figure S2: Binding mode of AgrA with positive control antivirulence compound savirin, staquorsin, and bumetanide using molecular docking. Here, AgrA protein is shown in the surface model with white grey color and antivirulence compounds in stick model, while the atom C, N, and O are shown in green, blue, and red color, respectively. (a) AgrA-savarin complex and (b) 2D interaction of savirin with AgrA residues. (c) Binding mode of AgrA-staquorsin and (d) 2D interaction of staquorsin with AgrA. (e) Binding mode of AgrA-bumetanide and (f) 2D interaction of bumetanide with AgrA residues. The antivirulence compound savirin, staquorsin, and bumetanide prefer a similar binding mode and interaction with AgrA residues as reported by the earlier studies.
